# Activators and Inhibitors of NRF2: A Review of Their Potential for Clinical Development

**DOI:** 10.1155/2019/9372182

**Published:** 2019-07-14

**Authors:** Natalia Robledinos-Antón, Raquel Fernández-Ginés, Gina Manda, Antonio Cuadrado

**Affiliations:** ^1^Instituto de Investigaciones Biomédicas “Alberto Sols” UAM-CSIC, Instituto de Investigación Sanitaria La Paz (IdiPaz) and Department of Biochemistry, Faculty of Medicine, Autonomous University of Madrid, Madrid, Spain; ^2^Centro de Investigación Biomédica en Red sobre Enfermedades Neurodegenerativas (CIBERNED), ISCIII, Madrid, Spain; ^3^Victor Babes National Institute of Pathology, Bucharest, Romania

## Abstract

The transcription factor NRF2 (nuclear factor erythroid 2-related factor 2) triggers the first line of homeostatic responses against a plethora of environmental or endogenous deviations in redox metabolism, proteostasis, inflammation, etc. Therefore, pharmacological activation of NRF2 is a promising therapeutic approach for several chronic diseases that are underlined by oxidative stress and inflammation, such as neurodegenerative, cardiovascular, and metabolic diseases. A particular case is cancer, where NRF2 confers a survival advantage to constituted tumors, and therefore, NRF2 inhibition is desired. This review describes the electrophilic and nonelectrophilic NRF2 activators with clinical projection in various chronic diseases. We also analyze the status of NRF2 inhibitors, which at this time provide proof of concept for blocking NRF2 activity in cancer therapy.

## 1. Introduction

**Figure 1:**
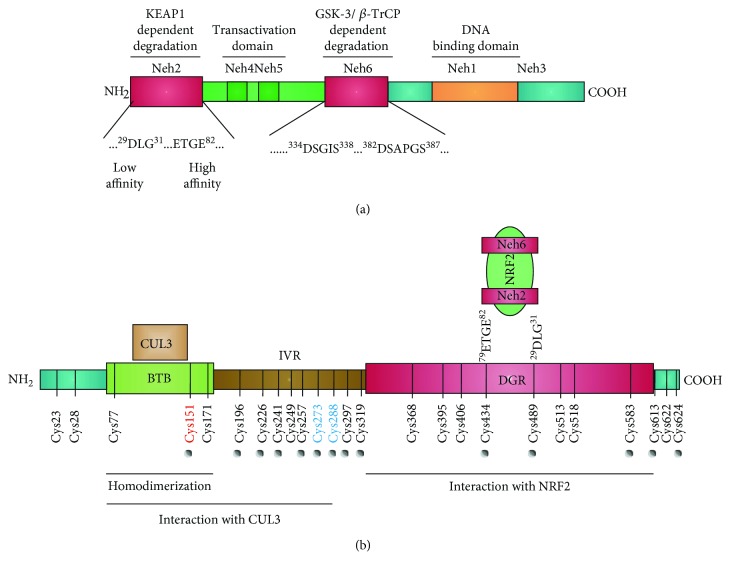
Domain structures of NRF2 and KEAP1. (a) Domain structure of NRF2. NRF2 possesses six highly conserved domains called NRF2-ECH homology (Neh) domains [167]. The functional role of each Neh domain is specified. Within the Neh2 domain, the low-affinity (DLG) and high-affinity (ETGE) binding domains to KEAP1 are zoomed in. (b) Domain structure of a KEAP1 monomer showing the position of cysteine residues. The N-terminal BTB (bric-a-brac, tramtrack, broad complex) domain participates in homodimerization and binding to CUL3/RBX1. The C-terminal region, DGR (double glycine repeat) domain, contains a double glycine repeat called Kelch repeat that binds NRF2-Neh2 domain. The intervening region (IVR/LR) connects BTB and DGR domains and is particularly rich in redox-sensitive cysteine residues. Red and blue cysteine residues in KEAP1 are the most relevant for electrophile reactivity. This figure has been modified and extended from [168] to highlight the degradation domains in NRF2 and the cysteines of KEAP1.

Nuclear factor erythroid 2-related factor 2 (NRF2) is the product of the *NFE2L2* gene and belongs to the cap′n′collar transcription factor family. By sequence homology with other orthologs, the domains termed Neh1-7 have been traditionally allocated in this protein (Figure 1(a)). At the C-terminus, NRF2 contains a basic leucine-zipper (bZip) domain that participates in the formation of heterodimers with other bZip proteins, like small muscle aponeurosis fibromatosis (MAF) K, G, and F [1, 2]. These heterodimers regulate the expression of about 250 human genes that present a regulatory enhancer sequence termed Antioxidant Response Element (ARE; 5′-TGACNNNGC-3′) and participate in multiple homeostatic functions including regulation of inflammation, redox metabolism, and proteostasis [3–6].

From a clinical perspective, it is of utmost importance that NRF2 can be targeted pharmacologically in diseases underlined by oxidative stress and inflammation, such as neurodegenerative, vascular, and metabolic diseases as well as cancer [7, 8]. In models of most chronic diseases, a reinforcement of homeostasis through NRF2 activators provides a beneficial therapeutic effect. In cancer, the pharmacological regulation of NRF2 appears to be context dependent. It is generally accepted that NRF2 inhibitors not only reduce the survival and proliferative advantage of cancer cells but also sensitize tumors to chemo- and radiotherapy [9]. In this review, we describe the pharmacological activators of NRF2 that are in several stages of pharmacological development for the treatment of several chronic diseases. The most developed compounds activate NRF2 by preventing its degradation by KEAP1-dependent mechanisms. We also discuss the current state of NRF2 inhibitors which may be highly relevant for cancer therapeutics although at this time they are still in early phases of development.

## 2. Physiologic Regulation of NRF2

NRF2 is ubiquitously and constitutively expressed by cells, thus ensuring their prompt protective response to oxidative, inflammatory, and metabolic stresses. Under normal physiological conditions, NRF2 has a rapid turnover and presents a half-life of about 20-30 min due to its constant degradation by the ubiquitin proteasome system [10, 11]. Therefore, under nonstressed conditions, low NRF2 levels provide basal expression of its target genes.

The main control of NRF2 stability is exerted by the E3 ligase adapter Kelch-like ECH-associated protein 1 (KEAP1). KEAP1 is a homodimer protein that comprises three functional domains (Figure 1(b)): a broad complex, tramtrack, bric-a-brac (BTB) homodimerization domain, an intervening region (IVR), and a C-terminal Kelch domain with a double glycine repeat (DGR). The Kelch domain binds to the Neh2 domain of NRF2 at two amino acid sequences: DLG and ETGE. Experiments based on isothermal calorimetry have led to the conclusion that the ETGE motif exhibits about one hundred times higher affinity for KEAP1 than the DLG motif [12]. KEAP1 presents NRF2 for ubiquitination by the E3 ligase complex formed by Cullin3 and RBX1 proteins (CUL3/RBX1) [13], resulting in subsequent NRF2 degradation by the proteasome 26S [2, 14].

KEAP1 contains 27 cysteine residues in humans, converting this protein in a redox sensor for endogenous and environmental oxidative signals as well as for electrophilic reactions [15]. Under redox-challenging conditions, the cellular redox buffers comprising glutathione (GSH), thioredoxin, etc. maintain low intracellular levels of reactive oxygen species (ROS) and glutathionylated proteins. However, ROS oxidize thiols and induce glutathionylation and alkylation of macromolecules, therefore having the capacity to modify KEAP1 cysteines [16]. From a pharmacological perspective, electrophile reaction with some cysteines of KEAP1 leads to the formation of adducts that prevent the ubiquitination NRF2, resulting in its stabilization, nuclear translocation, and transcriptional induction of NRF2-target genes [7, 8].

An alternative mechanism for proteasomal degradation of NRF2 is mediated by the glycogen synthase kinase 3 (GSK-3) and the E3 ligase adapter *β*-TrCP. GSK-3*α* and *β* are serine/threonine protein kinases involved in several signaling pathways such as receptor tyrosine kinase, WNT, and Hedgehog that influence cell division, survival, and development [17, 18]. GSK-3*α* and *β* are maintained in an inactive state under normal conditions due to their inhibition by AKT-mediated phosphorylation at their N-terminal pseudosubstrate domain or by sequestration in protein complexes. However, in the absence of receptor signaling, active GSK-3 phosphorylates NRF2 at the Neh6 domain (DSGIS). This phosphodomain recruits *β*-TrCP, which recognizes pSGIpS, and the CUL1/RBX1 complex for ubiquitin-proteasome degradation [19]. *β*-TrCP also recognizes another motif in the Neh6 domain of NRF2 (DSAPGS) which appears to be constitutively phosphorylated in a GSK-3-independent manner [20]. Additional degradative systems are able to regulate NRF2 at posttranscriptional level, such as the inositol-requiring enzyme (IRE1)/E3 ubiquitin ligase synoviolin (HRD1) [21].

NRF2 can be regulated at the transcriptional level. The *NFE2L2* gene promoter presents several regulatory sequences: (a) one xenobiotic response element (XRE; 5′-TA/TGCGTGA/C-3′) at -712 and two XRE-like sequences at +755 and +850 that are recognized by the transcription factor Aryl Hydrocarbon Receptor (AHR) [22]; (b) two ARE-like sequences at -492 (AREL1; TGACTCCGC) and -754 pb (AREL2; TGACTGTGGC), which allow NRF2 autoregulation [23]; (c) one 12-O-tetradecanoylphorbol-13-acetate-response element (TRE) (TGCGTCA) at +267 to +273 pb that is activated by the oncogenic KRAS [24], BRAF, and MYC [25] hence being critically involved in carcinogenesis; (d) one NF-*κ*B binding site that responds to inflammatory stimuli [26]; and (e) epigenetic changes such as promoter methylation, microRNAs including miR-144 [27], miR-28 [28], miR-98-5p [29], and long noncoding RNA deregulation [30] that contribute to changes in expression of the NRF2-coding gene.

## 3. Pharmacologic Activators of NRF2

**Figure 2:**
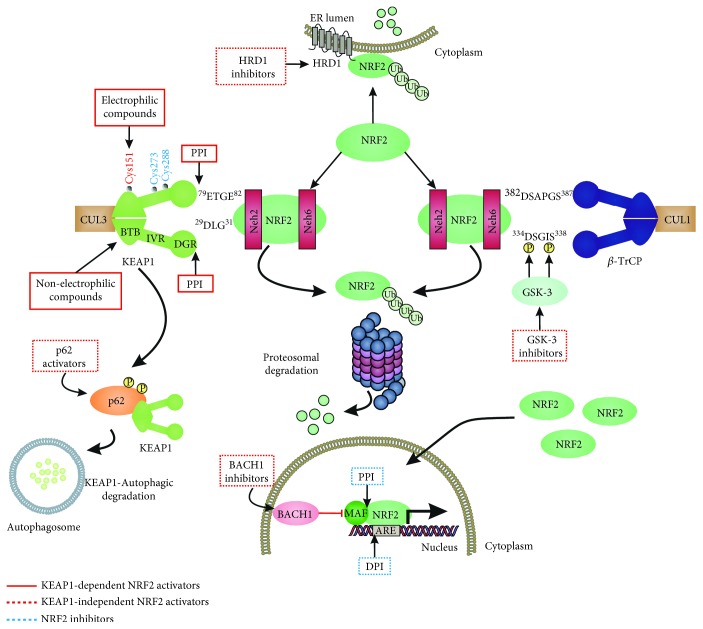
Summary of the pharmacological strategies to modulate NRF2 activity.

The so-called “NRF2 activators” should be more precisely termed “KEAP1 inhibitors” as their molecular target is in fact KEAP1 [31]. These compounds can be classified as electrophiles, protein-protein interaction (PPI) inhibitors, and multitarget drugs (Figure 2).

### 3.1. Electrophilic Compounds

Most pharmacological NRF2 activators are electrophilic molecules that covalently modify cysteine residues present in the thiol-rich KEAP1 protein by oxidation or alkylation [32–34]. Many cysteines of KEAP1 are modified by different electrophiles [35–37]. Cysteines Cys-151, Cys-273, and Cys-288 [38, 39] appear to be the most susceptible to electrophile reaction [40, 41]. Other sensitive cysteines are Cys-226, Cys-434, and Cys-613. This “cysteine-code” controls KEAP1 activity when the protective response mediated by NRF2 is needed. Selected electrophilic activators of NRF2 that are in various stages of clinical development are presented in Table 1.

One mechanism of KEAP1 inhibition is the sequestration in complexes with NRF2 that cannot be ubiquitinated. Modifications of several cysteines in KEAP1 generate a nonfunctional closed state with both Neh2 motifs (DLG and ETGE) of NRF2 interacting with the KEAP1 dimer but not leading to ubiquitination. As a result, free KEAP1 is not regenerated at a sufficient rate and newly synthesized NRF2 escapes KEAP1-mediated ubiquitination and subsequent degradation [42].

Another mechanism of KEAP1 inhibition is related to its interaction with the CUL3/RBX1 complex, required for NRF2 ubiquitination. Cys-151 located at the BTB domain influences the interaction of KEAP1 with CUL3. The crystal structure of the BTB domain bound to the pentacyclic triterpenoid 2-cyano-3,12-dioxo-oleana-1,9(11)-dien-28-oate (bardoxolone, CDDO, RTA401) indicates that adduct formation with Cys-151 most likely disrupts the interaction between KEAP1 and CUL3 [43–45]. As a result, KEAP1 is clogged in a NRF2 bound conformation, and newly formed NRF2 escapes ubiquitination. Synthetic triterpenoids have been derived from the natural compound oleanolic acid to provide them with strong Michael acceptor reactivity. This is achieved mainly through the addition of enone and ciano groups to the A ring and another enone group to the C ring [46, 47]. Bardoxolone methyl (CDDO-Me or RTA 402) reached clinical trials for the treatment of advanced chronic kidney disease (CKD) and type 2 diabetes mellitus [48]. Although phase II clinical trials demonstrated long-term increment in glomerular filtration, CDDO-Me was halted at phase III due to cardiovascular safety issues [49]. A new phase II clinical trial has recently started recruiting patients with rare chronic kidney diseases to better define the safety and efficacy profiles of CDDO-Me. Currently, CDDO-Me is also under clinical study for the Alport syndrome and pulmonary hypertension. In an effort to improve the safety profile, a second-generation difluoromethyl acetamide derivative of bardoxolone methyl, called RTA-408 (Omaveloxone), is now under clinical investigation in phase II clinical trials for Friedreich's ataxia, ocular inflammation, and pain after ocular surgery [50]. Recently, a preclinical study evaluated RTA-408 for diabetic wound recovery and pointed NRF2 upregulation as responsible for the observed improvement in regenerative capacity [51].

The most successful NRF2 activator to date is the fumaric acid ester dimethyl fumarate (DMF) (BG-12 or Tecfidera, from Biogen) that has been approved in 2013 by FDA for relapsing-remitting multiple sclerosis (MS) [52–55]. Previously, DMF was authorized for the treatment of psoriasis [56]. DMF was shown to reduce the number of peripheral T cells, CD8^+^ cells being more sensitive to DMF than CD4^+^ cells [57, 58]. DMF also reduces total B lymphocyte counts, especially memory B cells, along with a decrease in granulocyte-macrophage colony-stimulating factor, IL-6, and TNF-*α* production, leading to an anti-inflammatory shift in B cell responses [59, 60]. The DMF-induced activation of NRF2 in the central nervous system was described in the MS mice model of experimental allergic encephalomyelitis [61]. In this model, DMF-dependent NRF2 activation correlated with an improvement in the clinical course of MS, favored axon preservation, and increased astrocyte activation. These beneficial effects of DMF did not occur in NRF2-null mice, hence indicating that DMF was acting mainly by targeting the NRF2 pathway. DMF is mostly converted to monomethyl fumarate (MMF) by intestinal esterases, and only a small fraction is found in blood conjugated with glutathione [62]. Therefore, an oral formulation of a monomethyl fumarate (MMF) derivative, diroximel fumarate (2-(2,5-dioxo-1-pyrrolidinyl)ethyl ester; ALKS-8700; Alkermes) which exhibits improved bioavailability and efficacy, is currently under phase III trial for MS [63, 64]. However, the biological effects of these fumaric acid esters are not fully characterized and KEAP1/NRF2-independent effects are being described. For instance, it has been reported that DMF and MMF activate the nicotinic receptor hydroxycarboxylic acid receptor 2, which is expressed in immune cells and gut epithelial cells, resulting in NRF2-independent anti-inflammatory responses [65].

Oltipraz (4-methyl-5(pyrazinyl-2)-1-2-dithiole-3-thione) is a NRF2 inducer that enhances GSH biosynthesis and phase II detoxification enzymes, such as NQO1. Oltipraz is a promising chemopreventive agent [66] under phase III clinical trial for the treatment of nonalcoholic fatty liver disease.

Ursodiol (ursodeoxycholic acid) is an FDA-approved drug for the treatment of primary biliary cirrhosis. Although its cytoprotective mechanisms have not been elucidated yet, several research groups suggested that the upregulation of NRF2 by ursodiol induces detoxification and antioxidant mechanisms that play a role in its therapeutic efficacy [67, 68].

Several natural compounds have been identified as electrophilic NRF2 inducers, including sulforaphane, curcumin, resveratrol, quercetin, genistein, and more recently andrographolide [69]. For instance, sulforaphane (SFN), an isothiocyanate found in cruciferous vegetables, has been successfully used for the treatment of patients with type II diabetes mellitus [70, 71]. Due to the capacity of SFN to cross the blood-brain barrier, it protects against neurodegenerative disorders as demonstrated in murine models of disease. Regarding acute brain damage, SFN was shown to exert protective effects in hypoxic-ischemic injury in rats by reducing the infarct ratio and by upregulating NRF2 and HO-1 [72, 73]. In neurodegenerative disease models, SFN proved protective capacity against the neurotoxic A*β*_1-42_ peptide in neuronal cells [74]. *In vivo*, SFN ameliorated cognitive impairment in an acute mouse model of Alzheimer disease (AD) [75]. In Parkinson disease (PD), SFN protected dopaminergic cells against the cytotoxic effects of 6-hydroxydopamine [76]. In the 1-methyl-4-phenyl-1,2,3,6-tetrahydropyridine mouse model of PD, SFN counteracted astrogliosis and microgliosis and reduced the death of dopaminergic neurons [77–79]. To improve the stability of SFN, Evgen Pharma has developed a cyclodextrin formulation, SFX-01, which is under phase II clinical trial for the treatment of subarachnoid haemorrhage. A hybrid molecule of SFN and melatonin (ITH12674) was designed to have a dual “drug-prodrug” mechanism of action for the treatment of brain ischemia [80].

Another natural compound that modifies Cys-151 in KEAP1 and has also ROS-scavenging activity is curcumin, a linear diarylheptanoid present in turmeric (*Curcuma longa*) [81]. It has been used for the treatment of obesity, metabolic syndrome, and prediabetes [82–84]. Furthermore, curcumin has been shown to suppress the deleterious action of carcinogens by activating NRF2 [85, 86].

9-Nitro-octadec-9-enoic acid (OA-NO_2_) is a nitro-fatty acid with anti-inflammatory properties. OA-NO_2_ reacts with several cysteine residues of KEAP1, but mainly with Cys-273 and Cys-288, and its activity seems to be independent of Cys-151 [36]. CXA-10 (10-nitro-9(E)-octadec-9-enoic acid) is an isomer of OA-NO_2_ which has proven efficacy the uni-nephrectomized deoxycorticosterone acetate-high salt mouse model of CKD [87] and is under several phase I clinical trials for the treatment of this disease [88] and under phase II trials for the treatment of pulmonary arterial hypertension and primary focal segmental glomerulosclerosis.

The list of electrophilic compounds able to interact with KEAP1 is continuously growing. For instance, some compounds like 15-deoxy-*Δ*12,14-prostaglandin J_2_ interact with Cys-273 and Cys-288 of the KEAP1 homodimer [40]. This prostaglandin has a cyclopentenone core that is able to modify covalently Cys-273 and induce NRF2 in models of ureteral obstruction [89], hepatic ischemia-reperfusion injury [90], and atherosclerosis [91]. However, its clinical use is still far from being demonstrated. In a recent study, the metabolite itaconate was described as a NRF2 activator that alkylates cysteines 151, 257, 288, 273, and 297 of KEAP1. A cell-permeable itaconate derivate, 4-octyl itaconate, protects against lipopolysaccharide cytotoxicity, thus providing an anti-inflammatory response. Furthermore, this compound is a more potent NRF2 activator than DMF [92]. Some other examples are *tert*-butylhydroquinone [93], diethyl maleate [94], TFM-735 [95], and nitric oxide [96]. However, most of these compounds have not evolved beyond proof-of-concept experiments, and a long way needs to be covered to characterize their pharmacodynamic properties, clinical safety profile, and efficacy in noncommunicable diseases.

### 3.2. Protein-Protein Interaction Inhibitors of the KEAP1-NRF2 System

Protein-protein interaction (PPI) inhibitors interfere with the docking of NRF2 to the Kelch propeller of KEAP1 and provide more selectivity over electrophilic compounds which may eventually form adducts with redox-sensitive cysteines other than those in KEAP1 [97]. Based on the X-ray crystal structure of KEAP1 [98], small PPI inhibitors have been designed to impede the binding of the ETGE motif to KEAP1 [99]. The ETGE motif adopts a *β*-hairpin structure that docks to the Kelch propeller of KEAP1 through specific hydrophobic and electrostatic interactions [98, 99]. A similar strategy is devised to prevent the interaction of the low-affinity DLG motif which is required for correct lysine ubiquitination in NRF2 [13].

The first PPI inhibitors of KEAP1 were designed from a series of truncated NRF2 peptides [100, 101]. Some selected peptides are shown in Table 2. It was found that the minimal binding sequence of NRF2 required for docking to KEAP1 is the 9-mer sequence LDEETGEFL [100–102]. A related peptide was designed to increase cell penetrance by adding the Tat sequence of the human immunodeficiency virus and the cleavage sequence of calpain (-Cal-Tat). This peptide demonstrated neuroprotection and cognition-preserving effects in a mouse model of cerebral ischemia [103]. Moreover, hybrid peptides based on both the region of interaction between KEAP1 and NRF2 (ETGE motif) and with the region of interaction between KEAP1 and p62/Sequestosome-1 (SQSTM1) exhibited superior binding activity compared to either native peptide alone [104]. Due to unfavorable drug-like properties, such as low oral bioavailability and cellular permeability of peptides, research has been lately focused on the development of small molecules. However, a cyclic peptide was used recently to improve KEAP1 binding and NRF2 accumulation in cells [105].

Current PPI inhibitors are tetrahydroisoquinoline [97, 106], thiopyrimidine [107], naphthalene [108], carbazone [109], and urea derivatives [110]. Recently, the naphthalene-based nonelectrophilic PPI inhibitors were modified to develop nonnaphthalene heterocyclic scaffold based on 1,4-isoquinoline that avoids the carcinogenic and mutagenic properties of naphthalenes [111]. Some patents addressing these small molecules are presented in Table 3.

Several PPI inhibitors with improved selectivity over electrophiles have been identified through screening of small molecule libraries. These compounds include SRS-5, benzenesulfonyl-pyrimidone 2, N-phenyl-benzenesulfonamide, and a series of 1,4-diphenyl-1,2,3-triazole [106, 112–115]. Recently, a new protocol for identifying reversible modifiers of the NRF2/KEAP1 interaction was proposed [116]. The biochemical assays comprised time-resolved fluorescence resonance energy transfer as primary screening tool, surface plasmon resonance to evaluate the affinity of KEAP1 binders, and ^1^H-^15^N heteronuclear single-quantum coherence nuclear magnetic resonance assay to further analyze the binding mode. This protocol will help in identifying and improving the properties of reversible binders to KEAP1.

### 3.3. Other Mechanism of NRF2 Activation

The phosphorylation of NRF2 by GSK-3 leads to its ubiquitination by the E3 ligase *β*-TrCP and subsequent proteasomal degradation. An aberrant activity of GSK-3 is linked with several pathologies such as AD, cardiovascular diseases, or cancer among others [117–120]. Therefore, several clinical trials are now focused on GSK-3 inhibitors for the treatment of several pathologies [121]. For instance, the GSK-3-inhibitor Tideglusib, a thiadiazolidinone compound, was studied in phase II trials for AD in the ARGO study [122]. Another inhibitor is Enzastaurin which is intended for the treatment of solid and hematological cancers. Although Enzastaurin provided promising results at the preclinical level, treatment failed in phase II and III trials [123, 124]. GSK-3-dependent NRF2 phosphorylation was shown to be inhibited by nordihydroguaiaretic acid [125]. This compound and its derivative terameprocol are in phase I and II clinical trials for the treatment of several types of cancers, such as gliomas and leukemias (Table 4) [126].

Focusing on E3 ubiquitin ligase *β*-TrCP, it would be possible to develop small molecules able to disrupt the docking of NRF2 to *β*-TrCP, hence opening a new way regarding KEAP1-independent activators of NRF2 [127]. A novel E3 ubiquitin ligase linked to KEAP1-independent NRF2 degradation is HRD1 [21]. HRD1-dependent NRF2 degradation has been described in the context of cirrhotic liver. HRD1 is a transcriptional target of X-box-binding protein 1 (XBP1) that is upregulated upon activation of the inositol-requiring enzyme 1 (IRE1) during endoplasmic reticulum (ER) stress related to cirrhotic conditions. Inhibitors of HRD1 and IRE1 restore the NRF2 response in liver cirrhosis [21].

Several proteins contain a (E/S)TGE motif that resembles the high-affinity ETGE motif of NRF2. The motif confers to these proteins the ability to compete with NRF2 for KEAP1 binding, leading to a noncanonical mechanism of NRF2 stabilization [128]. Proteins containing the (E/S)TGE motif are dipeptidyl peptidase 3, Partner and Localizer of BRCA2, and SQSTM1/p62. SQSTM1/p62, a protein that transports specific cargos to the autophagosome, including KEAP1, sustains NRF2 stabilization and translocation to the nucleus [129–131]. Compounds which elevate SQSTM1/p62 levels, like rapamycin [132] and trehalose [133], are being therefore studied in several phase II and III trials in connection with diabetes mellitus, systemic lupus erythematosus, and autosomal dominant polycystic kidney disease.

Another way to inhibit the transcriptional activity of NRF2 is to impede its interaction with critical components in the nucleus. BTB domain and CNC homolog 1 (BACH1) is a transcriptional repressor which belongs to the cap′n′collar, b-Zip family. BACH1 competes in the nucleus with NRF2 to form heterodimers with small MAF proteins and blocks therefore the expression of ARE genes [134]. A recent study characterized the HPP-4382 compound as an inhibitor of BACH1 repression activity *in vitro* [135].

All these alternative mechanisms for NRF2 stabilization and activation suggest that a combinatorial pharmaceutical approach will be the best way to activate the cytoprotective responses mediated by NRF2.

## 4. Pharmacologic Inhibitors of NRF2

The implication of NRF2 in cancer is still controversial. Several studies described that NRF2 knockout mice are more susceptible to chemically induced carcinogenesis, pointing NRF2 as a potential tumor suppressor that limits carcinogenesis [136, 137]. On the other hand, NRF2 is overexpressed in many types of tumors, and it has been related to poor disease prognosis because it confers a survival and growth advantage to cancer cells, along with resistance to chemo- and radiotherapy [138–140]. Altogether, these results suggest a protective role of NRF2 in the first steps of cancer, but in advanced stages, NRF2 overexpression helps cancer cells to adapt to the tumorigenic demands. Cancer cells are “addicted” to NRF2 and resist treatment with chemotherapy or radiotherapy [141, 142]. Therefore, it is reasonable to assume that NRF2 inhibitors should sensitize tumor cells to anticancer therapies. In all cases, the mechanism of inhibition is either unknown or not specific, and therefore, NRF2 inhibitors are still far from being translated from bench to bedside.

### 4.1. Agonists of Nuclear Receptors

Ligands of the glucocorticoid receptor such as dexamethasone [143] and clobetasol propionate [144] inhibit NRF2 by blocking its transcriptional activity or preventing its nuclear translocation. All-*trans-*retinoic acid and bexarotene, agonists of the retinoic acid receptor-*α* and retinoid X receptor-*α*, inhibit the transcriptional activity of NRF2 [145, 146]. Retinoid X receptor-*α* appears to bind to the Neh7 domain of NRF2 preventing binding to the ARE enhancer [146]. The pharmacological value of this mechanism of NRF2 inhibition is limited by the multiple effects that are expected through the regulation of these nuclear receptors.

### 4.2. Natural Compounds

Several compounds of natural origin have been reported to inhibit NRF2. The quassinoid brusatol, extracted from *Brucea javanica*, inhibits the NRF2 transcriptional signature and sensitizes tumors and cancer cell lines to several chemotherapeutics [147]. However, its mechanism of action is not specific as it blocks protein translation, hence affecting other short-lived proteins as well [148–150].

The flavonoids luteolin [151] and wogonin [152] were reported to inhibit NRF2 and sensitize cells to anticancer drugs by increasing the instability to its transcript. However, later studies also indicated that these compounds may elicit NRF2 activation [153]. Therefore, their value as NRF2 inhibitor is highly controversial.

Other natural compounds such the mycotoxin ochratoxin A [154] and the coffee alkaloid trigonelline [155] prevent the nuclear translocation of NRF2. In leukemic cells, malabaricone-A, a plant-derived prooxidant, effectively inhibits NRF2 transcriptional activity as reflected by a reduction in HO-1 protein levels and leads to ROS accumulation and subsequent cell apoptosis [156]. Ascorbic acid, a well-known ROS scavenger, was found to sensitize imatinib-resistant cancer cells by decreasing the levels of the NRF2/ARE complex, hence reducing the expression of Glutamate-Cysteine Ligase Catalytic Subunit and dropping GSH levels [157]. In general, the main concern with these compounds is that their selectivity for NRF2 inhibition has not been conclusively demonstrated.

### 4.3. Other Approaches

The lack of knowledge about the fine structure of NRF2 hampers a straightforward strategy for the *in silico* analysis of small molecules that might dock to relevant domains of interaction with MAF proteins, ARE enhancer, etc. Therefore, a high-throughput screening was used which is helping in the identification of NRF2 inhibitors but still not providing selectivity [158]. A first-in-class compound, termed ML385, was found after the screening of a chemical library of 400,000 molecules. ML385 blocks NRF2 transcriptional activity and sensitizes KEAP1-deficient cells to carboplatin and other chemotherapeutics. ML385 interacts with the DNA-binding domain of NRF2 and most likely prevents the binding of NRF2 to AREs. However, given the similarity between AREs and other enhancers such as AP1, additional studies are needed to clearly establish if ML385 is selective for NRF2 or if it also inhibits other bZip transcription factors involved in chemoresistance.

Halofuginone, a synthetic derivate of febrifugine that is used in veterinary medicine, blocked the chemoresistance and radioresistance of cancer cells in parallel to the decrease of NRF2 protein levels [159]. It was found that halofuginone induces amino acid starvation resulting in global inhibition of protein synthesis.

Another compound, AEM1, decreased the expression of NRF2-controlled genes and sensitized KEAP1-deficient A549 lung tumor cells to various chemotherapeutic agents [160]. Although it seems that the anticancer effect of AEM1 is restricted to cell lines harboring mutations which render NRF2 constitutively active, the selectivity for NRF2 inhibition is not demonstrated yet.

In HeLa cells transfected with an ARE-driven luciferase reporter, a pyrazolyl hydroxamic acid, termed 4f, inhibited NRF2, reduced cell proliferation of myeloid cell lines, and increased apoptosis of acute myeloid leukemia cells [161]. Most likely, 4f altered the BCL2/BAX ratio and induced mitochondria-dependent apoptosis.

## 5. Conclusions

The NRF2/KEAP1 system represents a very promising pharmacological target to control common pathologic mechanisms of many chronic diseases characterized by low-grade oxidative stress and inflammation. A plethora of NRF2 activators, mostly of electrophilic nature, have been identified and a few are under clinical development. The pleiotropic effects of NRF2 on cell physiology together with potential off-target effects exerted by some NRF2 activators explain why drug development is moving slowly. The field of NRF2 inhibitors that may have a huge impact on cancer therapy is less advanced. Future work should be directed towards finding compounds with a good pharmacokinetic/pharmacodynamic profile for specific diseases.

## Figures and Tables

**Table 1:** Selected electrophilic activators of NRF2 under clinical development.

Compound	Type	Mechanism of action	Disease	Clinical trial	ClinicalTrials.gov identifier
Bardoxolone-methyl (CDDO-Me) 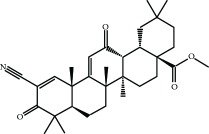	Synthetic triterpenoids	Electrophilic modification of KEAP1-Cys-151	Diabetic nephropathy	Phase II	NCT00811889
			IgA nephropathy CKD associated with type 1 diabetes Focal segmental glomerulosclerosis Autosomal dominant polycystic kidney	Phase II	NCT03366337
			Chronic kidney disease Type 2 diabetes Diabetic nephropathy	Phase III	NCT01351675
			Liver disease	Phase I/II	NCT00550849
			Hepatic impairment Healthy	Phase I	NCT01563562
			Advanced solid tumors lymphoid malignancies	Phase I	NCT00529438 NCT00508807
			Alport syndrome	Phase II/III cardinal	NCT03019185
			Pulmonary hypertension	Phase III RANGER	NCT03068130
			Pulmonary arterial hypertension	Phase III	NCT02657356
			Renal insufficiency, chronic Diabetes mellitus, type 2	Phase II	NCT01053936

RTA-408 (omaveloxolone) 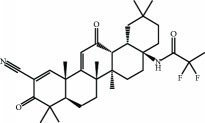	Synthetic triterpenoids	Electrophilic modification of KEAP1-Cys-151	Mitochondrial myopathy	Phase II	NCT02255422
			Friedreich's ataxia	Phase II	NCT02255435
			Inflammation and pain following ocular surgery	Phase II	NCT02065375
			Corneal endothelial cell loss Ocular pain Ocular inflammation Cataract surgery	Phase II	NCT02128113
			Melanoma	Phase I/II	NCT02259231
			Breast cancer	Phase II	NCT02142959

Dimethyl fumarate 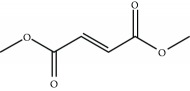	Fumaric acid ester	Electrophilic modification of KEAP1-Cys-151	Multiple sclerosis	*Approved*	
			Psoriasis	*Approved*	
			Rheumatoid arthritis	Phase II	NCT00810836
			Adult brain glioblastoma	Phase I	NCT02337426
			Cutaneous T cell lymphoma	Phase II	NCT02546440
			Obstructive sleep apnea	Phase II	NCT02438137
			Chronic lymphocytic leukemia Small lymphocytic lymphoma	Phase I	NCT02784834

ALKS-8700 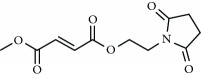	Fumaric acid ester (MMF-derivate)	Electrophilic modification of KEAP1-Cys-151	Multiple sclerosis	Phase III	NCT02634307

Oltipraz 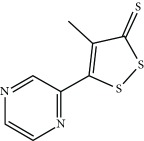	Organosulfur compound	Electrophilic modification of KEAP1-Cys-151	Nonalcoholic steatohepatitis	Phase III	NCT02068339
			Schistosomiasis	*Approved*	
			Lung cancer	Phase I	NCT00006457

Ursodiol 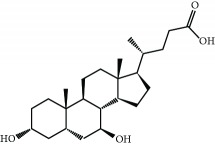	Biliary acid	Electrophilic modification of KEAP1-Cys-151	Cholestasis	Phase II/III	NCT00846963
			Diarrhea	Phase IV	NCT02748616
			Cholelithiasis	Phase III	NCT02721862
			Primary biliary cirrhosis	Phase IV	NCT01510860
			Barrett esophagus Low-grade dysplasia	Phase II	NCT01097304
			Chronic hepatitis C	Phase III	NCT00200343
			Type 2 diabetes mellitus	Phase II	NCT02033876

Sulforaphane 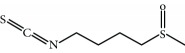	Isothiocyanate	Electrophilic modification of KEAP1-Cys-151	Schizophrenia	Phase II/III	NCT02880462
				Phase II	NCT02810964
				Phase II	NCT01716858
			COPD	Phase II	NCT01335971
			Atopic asthmatics	Phase I	NCT01845493
			Autism spectrum disorder	Phase II	NCT01474993
				Phase II	NCT02909959
				Phase II	NCT02677051
				Phase II	NCT02654743
				Phase I/II	NCT02561481
			Healthy	Phase I	NCT01008826
				Phase I	NCT02023931
			Melanoma	Phase I	NCT01568996
			Asthma	Phase I	NCT01845493
				Phase I/II	NCT01183923
			Prostate cancer	Phase II	NCT01228084
			Breast cancer	Phase II	NCT00843167
			Lung cancer	Phase II	NCT03232138
			Environmental carcinogenesis	Phase II	NCT01437501
			Alcohol sensitivity	Phase II	NCT01845220
			Aging	Phase II	NCT03126539
			Rhinitis, allergic	Phase II	NCT02885025
			Helicobacter pylori infection	Phase IV	NCT03220542
			Diabetes mellitus, noninsulin-dependent	Phase II	NCT02801448

Sulforadex (SFX-01) 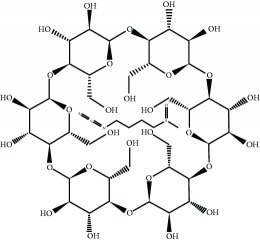	Sulforaphane/alpha-cyclodextrin complex	Electrophilic modification of KEAP1-Cys-151	Subarachnoid haemorrhage	Phase II	NCT02614742
			Breast neoplasm	Phase I/II	NCT02970682
			Prostate cancer	Phase I	NCT02055716 NCT01948362

ITH12674 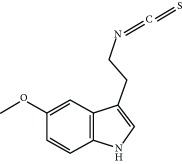	Melatonin-sulforaphane hybrid	Electrophilic modification of KEAP1-Cys-151	Brain ischemia	Preclinical PK	No clinical trials available

Curcumin 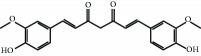	Stilbene	Electrophilic modification of KEAP1-Cys-151	Type 2 diabetes Prediabetes Insulin resistance Cardiovascular risk	Phase IV	NCT01052025
			Schizophrenia Cognition Psychosis	Phase I/II	NCT02104752
			Acute kidney injury Abdominal aortic aneurysm	Phase II/III	NCT01225094
			Chronic kidney diseases Diabetes mellitus, type 2 Polymorphism	Phase II/III	NCT03262363
			Alzheimer's disease	Phase I/II	NCT00164749
			Neoplasms	Phase II	NCT02944578
			Crohn's disease	Phase III	NCT02255370
			Chronic schizophrenia	Phase IV	NCT02298985
			Mild cognitive impairment	Phase II	NCT01811381
			Prostate cancer	Phase III	NCT02064673
			Major depression	Phase IV	NCT01750359

Resveratrol 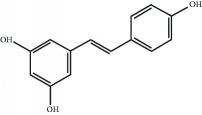	(*E*)-Stilbene derivate	Electrophilic modification of KEAP1-Cys-151	Type 2 diabetes	Phase I	NCT01677611
			Colon cancer	Phase I	NCT00256334
			COPD	N/A	NCT02245932
			Friedreich ataxia	Phase I/II	NCT01339884
			Nonalcoholic fatty liver	Phase II/III	NCT02030977
			Nonischemic cardiomyopathy	Phase III	NCT01914081
			Endometriosis	Phase IV	NCT02475564
			Chronic renal insufficiency	Phase III	NCT02433925
			Metabolic syndrome X	Phase II	NCT02114892
			Chronic subclinical inflammation Redox status	Phase III	NCT01492114
			Alzheimer's disease	Phase II	NCT01504854
				Phase III	NCT00743743
			Huntington disease	Phase III	NCT02336633

CXA-10 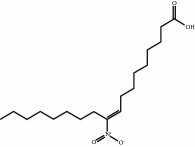	Nitro-fatty acid (NFA)	Electrophilic modification of KEAP1-Cys-273 and Cys-288	Acute kidney injury	Phase I	NCT02248051
			Pulmonary arterial hypertension (PAH)	Phase II	NCT03449524
			Primary focal segmental glomerulosclerosis (FSGS)	Phase II	NCT03422510

**Table 2:** Selected peptides acting as NRF2-KEAP1 protein-protein interaction inhibitors.

Sequence	Mechanism of action	Reference
LDEETGEFL-NH2	Binding to KEAP1-Kelch domain	[100, 101]
DEETGE-CAL-Tat (NH_2_-RKKRRQRRR-PLFAERLDEETGEFLPNH_2_)		[103]
Ac-DPETGEL-OH		[102]
FITC*β*-DEETGEF-OH		[102]
FITC-*β*-LDEETGEFL-OH		[102]
Ac-DEETGEF-OH		[102]
Ac-DPETGEL-OH		[102]
FITC-LDEETGEFL-NH_2_		[100]
FAM-LDEETGEFL-NH_2_		[108]
LQLDEETGEFLPIQGK(MR121)-OH		[107]
Ac-LDEETGEFL-NH_2_		[100, 101]
Ac-DPETGEL-NH_2_		[104]
Ac-NPETGEL-OH		[104]
St-DPETGEL-OH		[104]
YGRKKRRQRRRLQLDEETGEFLPIQ		[162]
c[GQLDPETGEFL]		[105]

**Table 3:** Selected small molecule activators of NRF2 acting as NRF2-KEAP1 protein-protein interaction inhibitors.

Compound	Type	Ref.	Patent
(SRS)-5 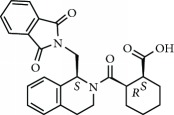	1,2,3,4-Tetrahydroisoquinoline core	[112]	WO2013/067036
Cpd 15 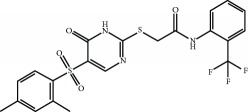	Benzenesulfonyl-pyrimidone	[107]	WO2016/202253
Cpd 16 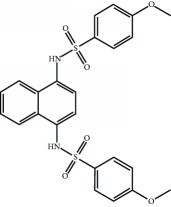	1,4-Diaminonaphthalene core	[107]	WO2016/202253
Compound 2 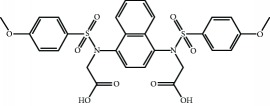	1,4-Diaminonaphthalene core	[163]	CN105566241A
3-(Pyridin-3-ylsulfonyl)-5-(trifluoromethyl)-2H-chromen-2-one (PSTC) 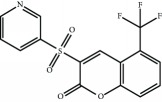	Sulfonyl coumarins	[164]	WO2015/092713
AN-465/144580 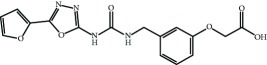	Other structure classes	[165]	JP2011/0167537
Compound 7 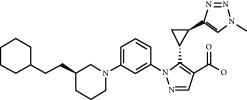	Arylcyclohexyl pyrazoles	[166]	WO2017060855

**Table 4:** Selected KEAP1-independent activators of NRF2.

Compound	Mechanism of action	Disease	Clinical trial	ClinicalTrials.gov identifier
Tideglusib 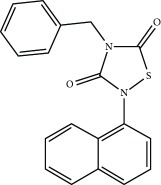	GSK-3 inhibition	Autism spectrum disorders	Phase II	NCT02586935
		Myotonic dystrophy 1	Phase II	NCT02858908
		Alzheimer's disease	Phase II	NCT01350362

Nordihydroguaiaretic acid (NDGA) 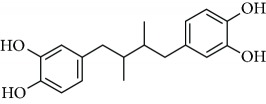	GSK-3 inhibition	Prostate cancer	Phase II	NCT00678015
			Phase I	NCT00313534
		Brain and central nervous system tumors	Phase I/II	NCT00404248

Terameprocol (NDGA derivative) 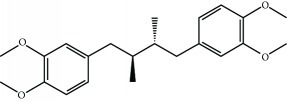	GSK-3 inhibition	High-grade glioma	Phase I	NCT02575794
		Leukemias Acute myeloid leukemia (AML) Acute lymphocytic leukemia (ALL)	Phase I	NCT00664677
		Refractory solid tumors Lymphoma	Phase I	NCT00664586

Enzastaurin 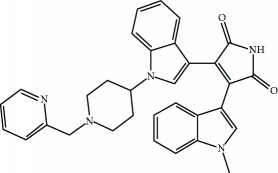	GSK-3 inhibition	Diffuse large B cell lymphoma	Phase III	NCT03263026
		Solid tumor Lymphoma, malignant	Phase I	NCT01432951

LS-102 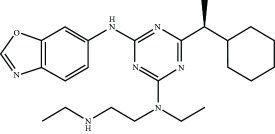	HRD1 inhibition	—	—	No clinical trials available

Rapamycin 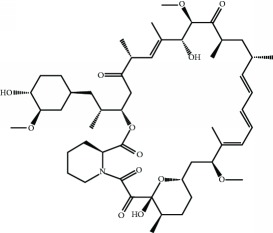	p62/SQSTM1 activation	Diabetes mellitus, type 1	Phase III	NCT01060605
		Systemic lupus erythematosus (SLE)	Phase II	NCT00779194
		Autosomal dominant polycystic kidney disease	Phase II/III	NCT00920309

HPP-4382 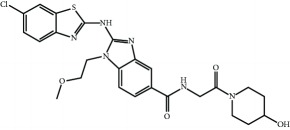	BACH1 inhibition	—	—	No clinical trials available

## Abbreviations

AD: Alzheimer's disease
AHR: Aryl hydrocarbon receptor
BACH1: BTB domain and CNC homolog 1
BTB: Bric-a-brac, tramtrack, broad complex
*β*-TrCP: Beta-transducin repeat-containing E3 ubiquitin protein ligase
CUL3: Cullin3
DMF: Dimethyl fumarate
DRG: Double glycine repeat
GSH: Glutathione
GSK-3: Glycogen synthase kinase
IVR: Intervening region
KEAP1: Kelch-like ECH-associated protein 1
MMF: Monomethyl fumarate
MS: Multiple sclerosis
*NFE2L2*: Gene encoding NRF2
NRF2: Nuclear factor erythroid 2-related factor 2
PD: Parkinson's disease
PPI: Protein-protein interaction
RBX1: RING-box protein 1
ROS: Reactive oxygen species
SFN: Sulforaphane
SQSTM1: Sequestosome-1
XRE: Xenobiotic response element.

## Supplementary Materials

Supplementary Materials Overview of the strategies aimed at pharmacologic regulation of NRF2. KEAP1-dependent and KEAP1-independent strategies to activate NRF2 are included in red boxes. The current strategies for inhibition of NRF2 are indicated in the grey boxes. PPI: protein-protein interaction inhibitor; DPI: DNA-protein interaction inhibitor.
