# Supplementary figures and images for: Activators and Inhibitors of NRF2: A Review of Their Potential for Clinical Development
Source: Oxid Med Cell Longev. 2019 Jul 14;2019:9372182. doi: 10.1155/2019/9372182 (PMC6664516; doi:10.1155/2019/9372182)

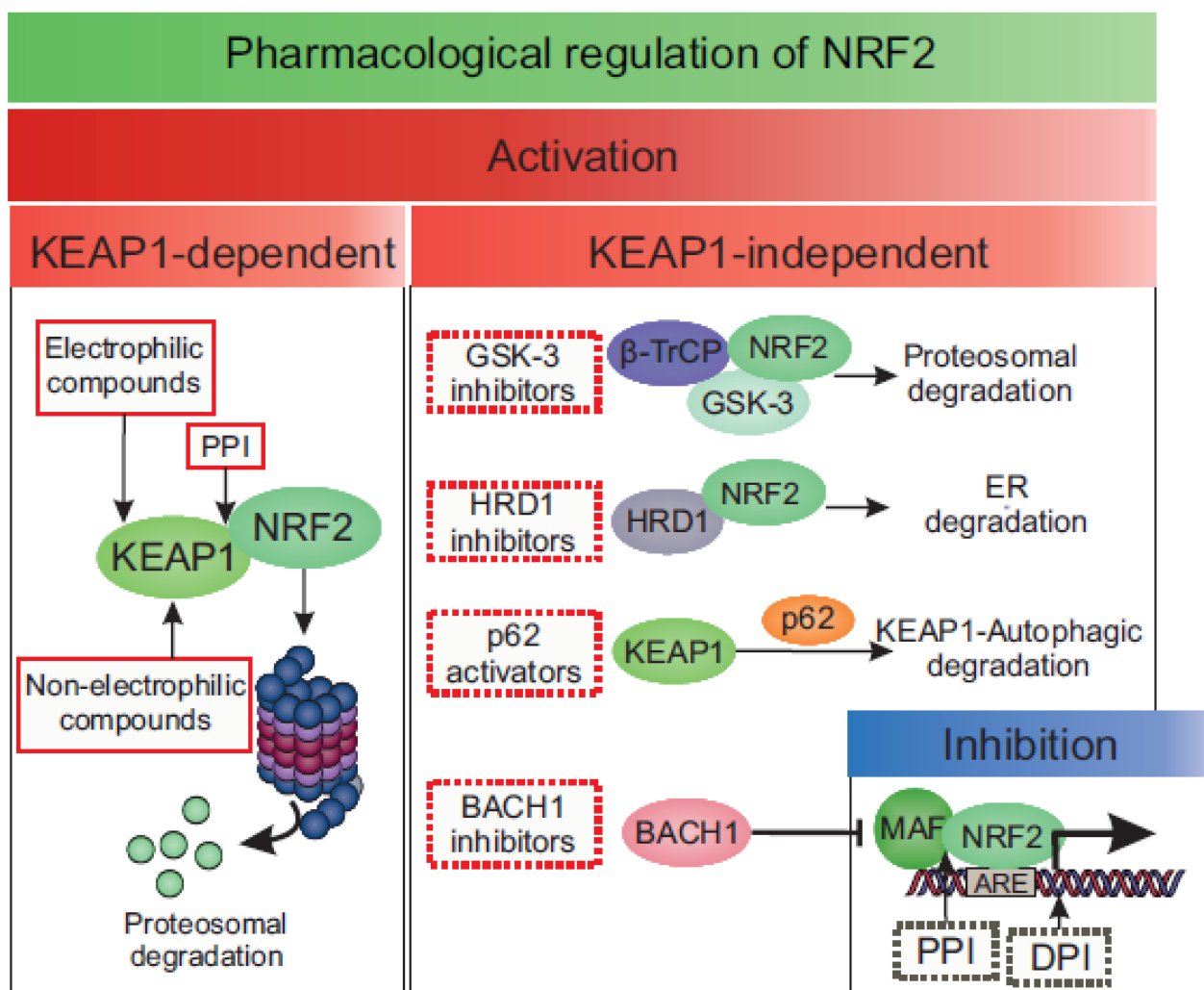

Graphical abstract

Supplement: Supplementary Materials — Overview of the strategies aimed at pharmacologic regulation of NRF2. KEAP1-dependent and KEAP1-independent strategies to activate NRF2 are included in red boxes. The current strategies for inhibition of NRF2 are indicated in the grey boxes. PPI: protein-protein interaction inhibitor; DPI: DNA-protein interaction inhibitor. [file 9372182.f1.pdf]
